# Health Support for a Remote Industrial Site

**DOI:** 10.3389/fpubh.2019.00180

**Published:** 2019-07-17

**Authors:** Thierry Lentz, Sabine Genty, Alain Gergereau, Alexis Descatha

**Affiliations:** ^1^AP-HP, EMS (Samu92) University Hospital of Paris West Suburb, Garches, France; ^2^FRANCE MÉDIAS MONDE, Issy-les-Moulineaux, France; ^3^AXA PARTNERS, Châtillon, France; ^4^AP-HP UVSQ, Occupational Health Unit, University Hospital of Paris West Suburb, Garches, France; ^5^Versailles St-Quentin University UVSQ, UMR-S 1168, Versailles, France; ^6^Inserm, U1168, UMS 011, Villejuif, France; ^7^Univ Angers, CHU Angers, Univ Rennes, Inserm, EHESP, Irset (Institut de Recherche en Santé, Environnement et Travail)—UMR_S1085, Angers, France

**Keywords:** medical engineering, occupational health, emergency preparedness, remote industrial site, Health Plan

## Abstract

This publication is derived from and rooted in the authors' experience in designing the Health Support of a remote industrial site. Summarizing the main steps of this design is the purpose of the approach. As a first step devoted to “Evaluation” (Chapters 1–5) are displayed the fundamentals for designing a Project Health Plan, such as a realistic and operative definition of “patient stabilization” and the principles of tactical reasoning for Medevacs, specifying how pathophysiology and logistic constraints should be correlated. A core element of the conceptual work consists in partnering these two domains, which usually each go their own way. Both should be considered in terms of delays: in life threatening situations, pathophysiology allows for a (maximum) delay before effective stabilization, while logistics dictates a (minimum) delay for reaching a stabilization facility. Ensuring that these two delays match is the desired result. Clearly, this conceptual work will unfold its full potential in low sanitary level countries, where most industrial commodities Projects take place, and where these delays are the longest. Next is detailed the audit/study preparation, i.e., the data gathering needed to get a clear picture of the Project conditions and concerns, workforce headcount and pattern, evacuation vectors and delays, and reference documents. Finally, risk assessment and a review of health facilities—in the vicinity and further away—complete the evaluation work. In a second phase devoted to “Implementation” (Chapters 6–9) is detailed how contracts with health providers, and health exhibits of industrial contracts should be conceived, and how on-site health support is designed, from the necessity of a pre-employment check to the design and organization of routine and emergency medicine facilities. Emergency preparedness and response plans, as well as medical coordination, should integrate with the HSE command chart. Overall, this document strongly advocates for joint engineering between HSE officers and medical specialists. An overview of key points for hygiene—often a separate topic covered in an offprint—is proposed. Finally, forward guidance for writing the audit/study report is proposed. This audit/study report must result in conclusive recommendations. Hence, a guide is proposed so that the report becomes a matrix of the Health Plan itself, and will be ended by a summary of findings and recommendations ready-to-use in Project management. In this way, the Health Plan will be launched, and gradually evolve and be amended as a “living document” throughout the lifetime of the Project.

## Introduction

### Remote Industrial Site Specific Features

Drilling for oil or gas, collecting ore deposits or having transformation plants closer to production units, often push commodity companies to more remote locations. At these remote industrial sites, in addition to the industrial unit itself, almost everything must be created: roads, airstrips, communication networks, power supply, accommodations, and of course health support.

To undertake such promethean projects, there is no other choice than to plan, prepare, schedule, map out, anticipate, and eventually engineer.

**Health support engineering** has long been sidelined in these gigantic organizations, dominated by technical engineers. However, the idea has surfaced that medical specialists could join Project teams and have their say in the global preparation processes.

### HSE and Security Counterparts

Health Safety and Environment (HSE) specialists are usually highly experienced technical engineers mainly devoted to the safety of all on-site work. Site firemen, First Aid responders, and the whole alarm and rescue chain are usually under their responsibility. When security is concerned, they team with adequate resources to build a Health, Safety, Security, and Environment (HSSE) supervisory capacity for everything from everyday camp life to complex industrial procedures.

The management of a medical case, obviously, will have to deal with these fields of competence.

This publication strongly supports very close cooperation between doctors and HS(S)E experts in the planning of reliable health support for any remote industrial site: the Health Plan methodology hereafter described is a procedure of **joint engineering** by a medical specialist and HS(S)E officers.

### Occupational Health vs. General Health Support

In developed countries, Occupational Health is a very comprehensive and well-developed domain of science, providing very precise recommendations, whereas health infrastructures (for rescue and hospitalization) are not a challenge. In remote areas, on the contrary, Occupational Health organization is usually poorly equipped, scarcely funded, and sometimes non-existent. Moreover, health facilities are often at a basic level.

Thus, the general health support for a Project situated in a remote area will encompass factors from industrial risk assessment and prevention, to very basic hygiene and epidemiological challenges. The design and implementation of a site clinic itself is an innovative act. With all these requirements for the health support of the workforce, it is a kind of **“Greenfield”** (ground-zero) **Occupational Health** that is needed, differing from the ordinary use of the term because it is multi-task and multi-purpose oriented.

### Necessity of an Audit or a Study

The joint engineering of a Project Health Plan, associating doctors and HS(S)E experts, is carried out just as any other engineering project is: detailed study produces observations, explores resources and methods, and must eventually coincide with management goals. Whether applied to a Greenfield Project or motivated by an existing health support assessment, the audit/study will have to **check, step by step, all the elements of care pathways**, for routine and acute cases. In low sanitary level countries, where most industrial commodities Projects take place, these care pathways may stretch to extended distances and evacuation delays.

This comprehensive study of a Project's surroundings is necessary in order to set up all the elements, providing what is specifically needed for the remote site conditions: **articulating pathophysiology and logistics** and **deploying them at strategical, tactical, and operational levels**.

### Aim

Though there are some documents available concerning certain issues relevant to building Health Support for a remote industrial site, there is no synthesis freely available or academic publication (to our knowledge).

Searching databases for “remote health” in academic publications returns essentially papers considering the aspect of the problem within the US (or developed countries), with topics such as telemedicine applications in rural health (1) or the promising use of drones (2).

With the significant exception of military health services developing the concepts derived from war medicine (3), and thus applicable to remote industrial sites as far as isolation is a common feature, topics specifically dedicated to Health Planning in remote commodities Projects are largely ignored so far. By the way, papers developing the most recent military experiences are essentially focused on Damage Control theorization and practices, which is integrated in our study, but is not the main focus of it.

After discussion of some fundamentals that should be known beforehand, we aim to detail the main steps for the evaluation (study preparation, risk assessment, evaluation of neighboring, and accessible health facilities). Then, we will detail the on-site Health Support implementation with the contracting strategy, and report/Health Plan writing (“after the audit”). The key points for hygiene will be summarized thereafter.

All these methods and conceptual works are not a synthesis of academic literature and, consequently bear a low quality of evidence. However, Evidence-Based Medicine and evidence-based decision making may not go without the recounting of clinical expertise as a significant part of the sources to consider (4, 5).

This kind of expertise of the sort, applied to Health Planning for remote industrial locations, is the contribution we intend to expose here, and must be seen as a first proposition for the topic.

As this publication is perhaps one of the first attempts to bring to the academic circles the making of a task that has been so far somewhat concealed in industrial think tanks, we fervently expect other publications to take up and evaluate every concept proposed in this publication.

## Fundamentals for Designing a Health Plan

Before describing the main evaluation steps, different definitions, and concepts should be noted.

### Strategy, Tactics, and Concepts of Operations

The Health Support of a Project has to be implemented at three levels: strategic, tactical, and operational.

- **The strategy** is displayed in the **Health Plan**. This is a “living” document, updatable, and amendable. It represents the largest view of a Project Health Support, covering every health-related topic, from Intensive Care to Occupational Medicine, including Hygiene, legal, and contractual issues, etc.
  Keeping this document “living” (updated and amended), is generally the responsibility of the **Chief Medical Officer** of the Project, or the **corporate doctor(s)** of the Head Company.
  The purpose is to keep the health support consistent with Project evolution and the management goals at all times.
  To fully coincide with Project management, the Health Plan must rely on studies, started at the same time preliminary studies are being conducted, at the very beginning of the Project itself. This phase is usually called: conceptual studies. A medical input at this early phase is necessary, though seldom performed, and is a warranty that no major gap will appear later on. As engineers, financial decision makers, legal specialists, and other cross competences start to draft the Project, a medical specialist must join the team and work hand in hand, usually close to HSE specialists.
  Pursued all along the Project's lifetime, this permanent medical elaboration and input is the very meaning of **Project Medical Engineering**.
  The core part of the Health Plan document is a tactical reasoning from which are inferred Medevac routings and a Care Organization System (see below) are inferred.
- **Tactics** are the result of reasoning, a train of thought integrating contingent parameters, and producing decision-making.
  In military practice, tactical reasoning is usually articulated as:
  ○ What are my enemies or unfavorable conditions?
    and
  ○ What are my friends or favorable conditions?
    and
  ○ What are my objectives?
    then
  ○ Knowing all that:
    ■ What do I need?
    ■ How do I proceed?
  In the context of remote industrial Project Health Support, the objective is to provide everyone with the best possible health care. “Everyone” being too unspecified, it is necessary to distinguish:
  ○ **Routine medicine** vs. **Emergency and Intensive Care** cases;
  ○ **Single patient** vs. **mass casualties**' situations;
  At a glance, considering remoteness of the industrial site, distances (and hence transport delays) to hospitals and other healthcare facilities clearly represent an unfavorable condition. On the other hand, site clinics and transportation vectors provide enhancement to the situation.
  Unraveling the situation obviously starts with **identifying** precisely which **healthcare facility(ies)** is/are useful in the vicinity of the Project location, **considering transportation vectors** available, and eventually **measuring the time** needed to get there.
  **Routine medicine** cases usually find a solution through treatment at site healthcare facilities, and identification of available external para-clinical facilities, drug and consumable procurement, and referrals.
  In contrast, **Intensive Care** cases are subject to the pressure of time.
  The reasoning will have to produce the best routing for every category of life-threatening situation.
  This is where pathophysiology and logistics interact.
  To solve this, a key concept is important: **stabilization** (the precise meaning of this word will be clarified in section What Stabilization Actually Means, as it is frequently misused). Introducing the stabilization concept, the challenge is to answer the question:“How fast and to what location should we evacuate a patient presenting such a critical clinical situation and—if relevant—after what treatment on site?”
  Matching the demands of pathophysiology and logistics is the necessary task, described in section Pathophysiology and Logistics.
  The results of the tactical reasoning for both routine medicine and Intensive Care cases are summarized in the table “**Care Organization System.”**
- **Concepts of Operations** (Primary Plans) is another wording derived from military terminology. It sets up procedures and plans describing the actions of all parties involved, in response to a pre-defined situation. It is the third level of implementation of Project Health Support. As a matter of fact, once the tactical reasoning is completed, some action needs will recur frequently, common to quite a few operational situations.
  “Who takes what actions in response to this pre-defined situation” is the key question for a Concept of Operations. This may be also labeled as a “**Primary Plan.”**
  As examples, Primary Plans may be composed for:
  ○ Ground transportation of a patient to the airport of …/to hospital…;
  ○ Call for a helicopter Medevac from the site to …;
  ○ Patient referral to hospital … for tests or investigations such as x-rays or lab work;
  ○ Patient care in clinic … under a given healthcare provision contract …;
  ○ …
    These plans should cover the entire range of duties involving the roles and responsibilities of, for example, the:
    ■ HSE manager;
    ■ Site Manager;
    ■ Patient's employer–in case of a sub-contracted worker;
    ■ Doctor on site–if any–;
    ■ Medics;
    ■ First Aid Responders;
    ■ …
    As well as the first contacts with an assistance company, or healthcare provider (see section Medical and Assistance (Evacuation) Providers), or next of kin, etc…
    Some other Primary Plans describe the very basic unfolding of the emergency response to a pre-defined risk, such as:
    ○ Injury of a worker at site zone…;
    ○ Fire in a workshop …;
    ○ Traffic accident on site road …;
    ○ Welding eye injury of a worker;
    ○ Acute intoxication to chemicals at work;
    ○ …
    These basic Primary Plans should cover the duties and actions of the entire site rescue chain:
    ■ Fellow workers without First-Aid capabilities and training;
    ■ Fellow workers with First-Aid capabilities and training;
    ■ Professional Site rescuers or firemen;
    ■ HSE manager;
    ■ Site medics;
    ■ Site nurse–if any –;
    ■ Site Doctor–if any –;
    ■ Ambulance driver;
    ■ …
    As well as the precise **report pattern** of by-standers to the HSE officer or medics or site doctor.
- **Other Plans**
  The label “**Contingency Plan**” is in most cases used to name an inventory of existing material and human resources relevant to Health Support. Though very useful for Health Support management, Contingency Plans considered in this way are framework documents and not operational plans.
  Finally, with some site management teams, the Primary Plans are gathered in a larger document called **Emergency Response Plan** or **Incident Management Plan**. The design of the global emergency response (i.e., industrial safety procedures) may vary from site to site, as well as the document(s) in which they are displayed.
  Whatever the presentation, the medical (health related) part of it—Medical Emergency Response Plan (MERP) must derive from a methodology addressing the three levels of organization described in this paragraph and integrating the **pathophysiology/logistics matching reasoning**.

### What Stabilization Actually Means

One clinical feature is common to all life-threatening clinical situations: A **self-degradation** through auto-feedback loops with a chain of cascading failures.

A **stabilization** facility is a medical structure that has the effective means to stop the self-degradation of the pathophysiology.

To identify what capacities and equipment of such a structure are needed, it is necessary to consider four different pathophysiological features:

- Traumatisms without head injury;
- Traumatisms with head injury;
- Acute cardiac disorders;
- Medical Intensive Care cases.
  Head injuries are here set apart from other injuries, since neurosurgery is a specific capability, sometimes missing in an otherwise fully equipped trauma center.
  Acute cardiac disorders are in most cases (considering industrial site workforce populations) related to an Acute Coronary Syndrome.
  Medical Intensive Care cases consolidate all non-surgical life-threatening situations that may occur under conditions such as: acute malaria, poisoning, septic shock, acute dehydration, and metabolic disorders, acute respiratory disorders, etc.
  Given this partition, the necessary and sufficient capacities and equipment to stop the self-degradation are displayed in the table below:

**Table:** 

	**Minimal**	**Desirable**
Trauma without head injury	Visceral and orthopedic surgery	CT Scan—Embolization
Trauma with head injury	Idem + CT scan and Neurosurgery	MRI—Embolization
Acute Coronary Syndrome	Cath Lab angiography^*^	
Medical Intensive Care cases (non-surgical)	Medical Intensive Care Unit^*^	
Stroke	MRI—Neurovascular Intensive Care Unit	

* *Routine laboratory tests are assumed to be part of the minimal capabilities of such facilities*.
  The “minimal” column displays what is necessary and sufficient for life-saving treatment, i.e., **stopping the self-deterioration**. A secondary evacuation to a better equipped structure may then be organized for completion of the treatment if, as mentioned, the initial treatment is minimal. But, once the pathophysiology deterioration stops, the patient is, by the actual meaning of this term, **stabilized**.
  Trauma cases are the epitome of this approach: the concept of **damage control surgery** is today widely accepted and practiced, notably by military surgeons. As a matter of fact, in many developing (or emerging) countries, local hospitals have only limited surgical capabilities and an inappropriate level of hygiene. One could say that damage control surgery is what they routinely do, since they lack sophisticated surgical capabilities. And that is precisely what is needed in the case of a remote industrial site for damage control. Hence, it is clearly preferable to first evacuate a trauma patient to such a closely localized and basic local hospital, and then, have a secondary evacuation to a more better equipped structure. Once, for example, internal abdominal bleeding is stopped, there is plenty of time (compared to the initial condition) to organize this secondary Medical Evacuation (Medevac).
  **Identifying and listing stabilization facilities**, as here defined, that are the closest to the industrial site, and measuring the time it takes to reach them, are the initial preparatory tasks in drafting the **tactical reasoning for Medevacs**.
  Unlike trauma cases, **cardio-vascular acute failures** may require a stabilization facility **very far from site**, often out of the country itself. In Africa, for example, recommendable Cath Labs are very few, and the study of evacuation delays may show that it is preferable to send the patient directly to Europe, South Africa, the Canary Islands or the UAE, which may in itself be a quasi-repatriation.
  Hence, the tactical reasoning for Medevacs in acute cases will probably generate **different routings**, depending on which of the four main pathophysiological features patients present with.

### Tactical Reasoning for Medevacs

#### Onset-to-Treatment Interval

For emergency treatment, the reasoning is based on the notion of the **Onset-to-Treatment Interval** (OTI) which is defined as the maximum acceptable length of time (given the pathophysiology knowledge) between the occurrence of the trauma/illness and the initiation of the effective life-saving treatment, as defined above.

This concept was introduced by Pr. P. Huguenard in his Disaster Medicine teaching, termed (in French) “*Intervalle libre thérapeutique*” (6, 7).

At any moment of patient care, the decision to be made by the chief doctor concerns whether the patient needs further stabilization, and where and how to get there, and is based upon patient clinical status.

#### Pathophysiology and Logistics

For each of the four clinical features defined above, the OTI will have to be compared with the evacuation delay needed to reach the adequate stabilization facility.

Considering a remote industrial site, the **OTI** usually tends to be **too short** whereas the **evacuation delay** tends to be **too long**.

To have these two delays matching, or getting as close as possible, is the purpose of the medical engineering work, to be performed **jointly by the medical specialist and the Project HSE officer**. It comprises:

- Reducing evacuation delays;
  AND
- Extending the OTI.
  To achieve this, factors which it is possible to deal with are:
- Faster transportation;
- Sophistication of primary care.
  ○ **Faster transportation** obviously makes the patient reaching the referral facility faster. Its total time must be properly estimated, **“all-inclusive,”** i.e.,: the transport itself, plus the time required for patient transfer from clinic to ambulance, ambulance to aircraft, ambulance to Emergency Room etc…This last part of the total transport time can be lengthy if the hospital lacks an adequate emergency structure.
    The impact of medical input from the very beginning of Project conceptual studies is striking here: when tracks, roads, airstrips, etc. are foreseen all along the industrial design (particularly for a greenfield Project) it is very important to consider also the usefulness of the transportation pattern for medical evacuations. A later revision of such infrastructure work would be very costly. In addition, when considering contracting a transportation provider (e.g., helicopter), keeping in mind Medevac capabilities (flight range and lifted load) is an obvious way to prevent further budget overspending.
  ○ **Sophistication of primary care** can be translated into **onsite pre-hospital Intensive Care** and **medical escort**.
    **Onsite pre-hospital Intensive Care** will slow the ongoing damage leading to a critical clinical situation and hence, extend the OTI. This can be fairly complicated, involving technical actions requiring a high level of skills and experience, even the ability to perform a general anesthesia (e.g., in case of a serious head injury). Site health personnel will have to be chosen from the entire range of available skills: Emergency doctors or intensivists, anesthetist nurses, emergency nurses, advanced life support medics… Usually, highly skilled health personnel are expatriates.
    Here again, what may appear costly at first glance, will actually be a substantial saving when a life-threatening situation occurs: hastily rearranging, at the last moment, for high-level medicalization capabilities will undoubtedly cost a lot, and may not properly work.
    From this, an important rule emerges concerning onsite health support implementation that will be detailed in section Routine Care Health Personnel and Emergency Medical Professionals: while **routine medicine** tailoring is conditional on the **volume of expected daily activity** (linked to workforce size and occupational risks), decisions about **emergency onsite medical capacities** depend on the **remoteness of the site and potential evacuation delays**. The larger the mismatch between OTI and evacuation delays, the greater the need for highly skilled health personnel.
    **A medical escort** while in transit has the same utility (extending the OTI) and is obviously necessary when sophisticated primary care has been carried out onsite, to avoid any discontinuity. The problem here is making the right decision as to whether the health personnel of this escort are to be withdrawn from the on-site medical team (implying that enough health personnel will stay onsite for continuous support), or outsourced from an external provider (with the ensuing time needed for their inbound transportation).

### Care Organization System

The result of the study, synthesizing the data from pathophysiology with evacuation delays and conditions from site, is displayed in a table called **Care Organization System**, displaying destinations for:

- Routine medicine complements (x-rays, biochemistry, other external para-clinical facilities, drug and consumable procurement, identified referrals)
- Stabilization facilities and how to get there, with or without a medical escort, etc.

Healthcare capabilities specified under the “minimal” category are missing in some remote area health facilities, if one considers only the nearest ones of these facilities. But, encompassing the whole country or sub-region, these capabilities will be found far away, sometimes very far. This is precisely what founds the need for a tactical reasoning prior to establishing Medevac routes.

In fact, when a given treatment capability is missing in the commonly accepted regional backup hospital, there comes up a difficult task for the doctor/HSE officer tandem elaborating Medevac flow charts: having the management accepting that a given category of patients must be sent to … here instead of … there. That is where astute and creative combination of logistic means is required to achieve an evacuation to the adequate facility, matching the contradictory requirements of pathophysiology and logistics.

### Medical and Assistance (Evacuation) Providers

For critical clinical cases, the last step in creating a pathway for stabilization facilities, is to indicate which company or provider will be responsible for each section of the route.

The subtleties of medical service contracting and provision are detailed in section Contracting Strategy: Subsidiaries, Contractors, and Subcontractors Personnel Health Support.

Usually, there will be:

- **A primary care provider**, present onsite, which may either be the head company itself, or an industrial contractor, or contractual medical provider. It is responsible for the evacuation's first leg;
- **A secondary care provider**, coming up at a time when it is scheduled to be dealt with, from a point defined as **a handover location**. The secondary care assistance provider usually operates from a logistic platform of general use for evacuations;
- **A handover location** is where responsibility of one Tier is transferred to another.
- **Different transportation legs**
  If the patient is an expatriate, the Medevac:
  ○ May be followed by a repatriation for hospitalization—in a second leg after stabilization;
  ○ May be a—first leg—repatriation if the relevant stabilization hospital with modern-level standards of medicine is in the developed country from which the patient originates.

For the patient to reach the stabilization facility is the focal point for the Chief Medical Officer (CMO), coordinating appropriate field (intensive) care, and transportation(s), should the patient's route be through different handover locations, relying on a chain of different assistance/healthcare providers.

### Flow Charts

**Figure 1:**
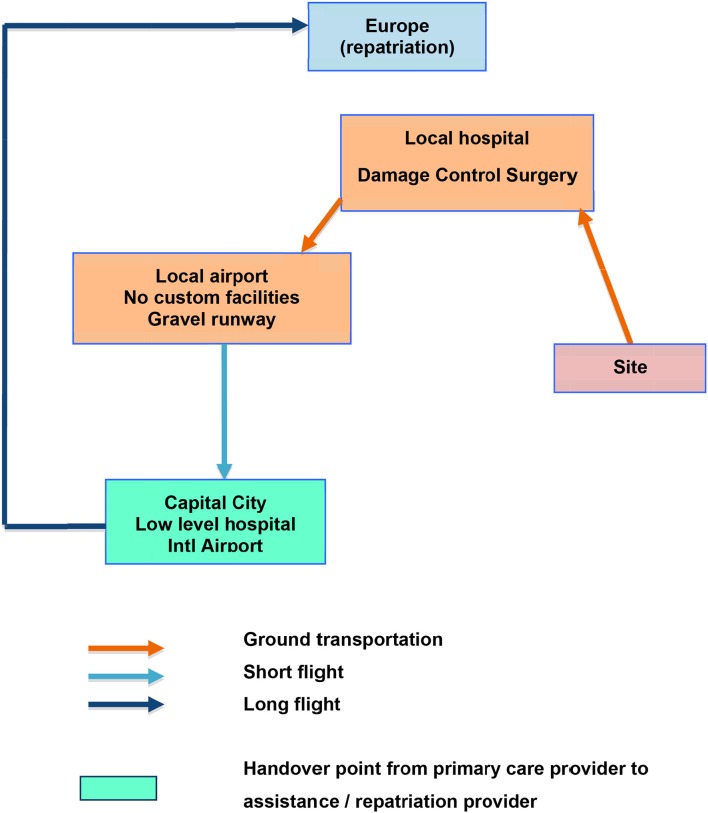
Example of possible flow chart for damage control (trauma patient).

**Figure 2:**
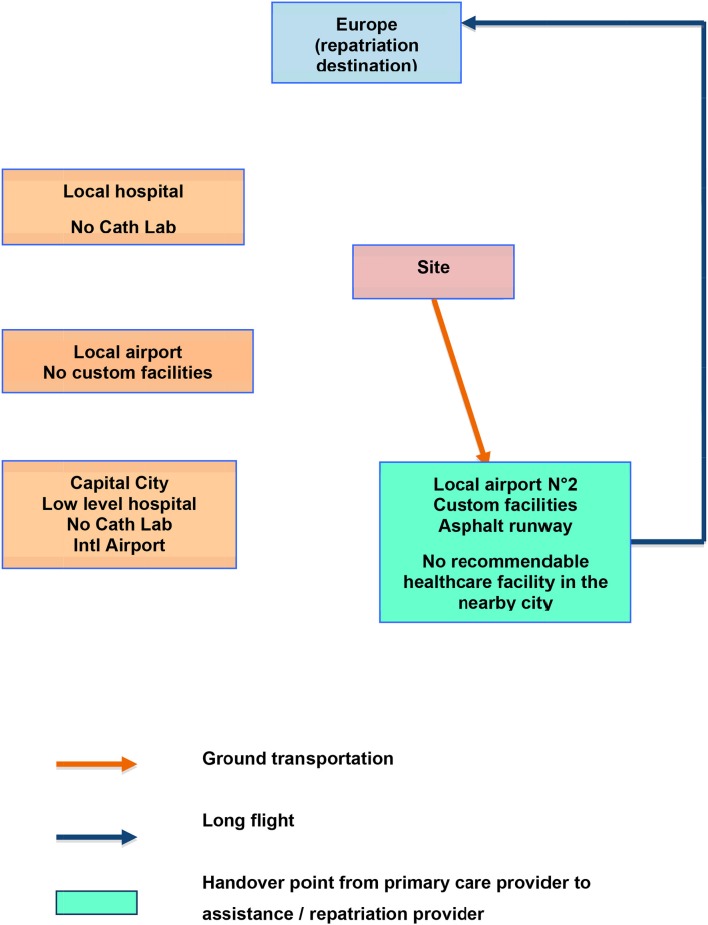
Example of possible flow chart for acute coronary syndrome.

Below are two examples of flow charts, supposed to feature two different clinical cases (trauma and acute coronary syndrome) from the **same industrial site** in Africa (Figures 1, 2).

The first one addresses a **trauma patient** for whom **damage control surgery** is performed at the nearby **local hospital**, after a short ground transportation. Then, the patient being stabilized, **a first leg evacuation** is organized through the nearby local airport. This one having no custom facility, a short flight with an aircraft able to land on a gravel runway brings the patient to the capital city. There, a **handover** will be made with the assistance/repatriation company after considering a stopover at the country's main hospital. It being of an inappropriate level, a **direct wing-to-wing transfer on the tarmac** of the airport would be the better choice, avoiding additional transfer delays.

From the same site, if the patient is an **expatriate** presenting with an **Acute Coronary Syndrome**: the local and capital hospitals would not be useful for stabilization, since they lack a reliable Cath Lab. But, exploring the surroundings, **another airport, 2**, is found further away, where there is **no healthcare facility**. This one has an asphalt runway and can accommodate **business jets**. Though the drive to get there is longer than that to the previous local airport, the tactical reasoning based on the pathophysiology of Acute Coronary Syndromes shows significant spared time and myocardial function with the following sequence:

- On site thrombolysis;
- Medical escort to airport N° 2;
- Direct evacuation by Air Ambulance (long haul flight) to the nearest center of modern capabilities. This may be, at the same time, a repatriation.

The flow charts should be spatially oriented even if distances are out of proportions. In our example which is located in Africa, Europe is at the top of the chart (to the North), while the damage control facility is North-West of the site and airport number 2 is South-East.

To complete what has been described above, the first part of designing the Health Support for a remote industrial site consists in additional evaluations, including audit preparation, risk assessment, and evaluation of existing health facilities—in the vicinity and further away.

## Audit/Study Preparation

### Project Situation and Concerns

Since medical input to be integrated into the Emergency Response Concept of a Project may be requested at any time, there is usually one of the following situations obtaining:

- An anticipated significant **change in Project scale** or development, which will lead to the existing health organization becoming overwhelmed or inadequate. This occurs frequently at mining sites, as the operation develops;
- A **change of the Project management** team. The new manager may want a reset of the existing Health Plan, or a comprehensive view of health-related issues;
- **A Greenfield Project**. Every industrial Project located in a remote area should benefit from medical engineering from the very beginning of the conceptual studies. However, this is not usually the case. Such a start-up plan as having the HSE officer and the Doctor drafting together, and very early on, the Emergency Response and a first Health Plan, is cost-effective, smart, and the best way to prevent potential failures. They will, as well, have to rethink the work along successive phases.
- **Recent health organizational issues**, notably concerning a Medevac not being executed properly, or unusual medical activity volume or expenses.

The first task of a medical engineer mandated to work on a Project Health Plan is to **understand what motivated the request**. This context is not systematically delivered in the wording of the Terms of Reference (section Audit/Study Terms of Reference).

In addition, the **preferred contact(s)** must be identified. An HSE officer is always one of them. It remains to be seen, however, whether this is at the head office or onsite or both. In large Projects, the HSE organization chart may be somewhat complex.

### People on Site

“**People on Site**” indicates those who are assigned onsite and their particular location. The medical aspects impacting Project personnel are labeled under “**Population(s) at Risk**” (in the section on Risk Assessment).

The goal here is to provide a clear view of personnel categories and status, and personnel locations, through the various phases of the Project.

**The breakdown of workforce components** is also called: **mobilization curve**, displaying who is in the workforce.

It is easy to distinguish between **expats** and **nationals**. These may be on the payrolls of the **head company**, **contractor**s, or both.

**Third Country Nationals (TCNs)**, however, represent an extremely heteroclite group. These workers are most often hired by contractors or sub-contractors and sourced from low labor-cost countries/developing countries. *Stricto sensu* they are expats. But the usually harsh conditions under which they are recruited, accommodated onsite, paid, and granted a right to healthcare and Medevacs, makes them a distinct category, on which special attention needs to be focused. Unpreparedness for emergency healthcare concerning these personnel is liable to lead to the greatest number of mishaps related to critical clinical situations.

**Figure 3:**
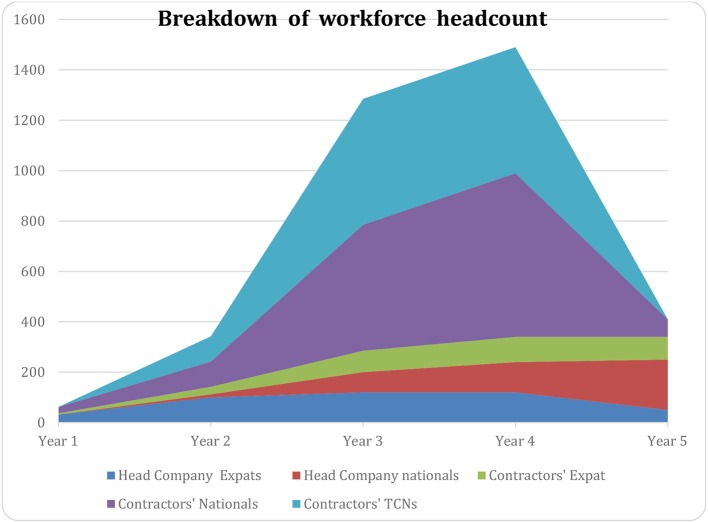
Example of the variation of breakdown of workforce during time (X axis: time, Y number of persons on site).

An example of the variation between these different groups is given in Figure 3:

The workforce chart for a large industrial Project is often teeming with a **cascade of subsidiaries, contractors, and sub-contractors**. Though tedious to implement and study, it is very important to have a clear picture of the details, as comprehensive as possible.

This will indicate, at the time of writing, the **health exhibits of the industrial contracts**, provided this job has been completed before the final contracts are formulated. Section Contracting Strategy: Subsidiaries, Contractors, and Subcontractors Personnel Health Support is devoted to the **Contractual Strategy**.

Given the **geographical distribution** of the workforce, another curve/chart needs to be drawn. It will also indicate the relative risks of injury following the Risk Assessment (section Risk Assessment) which details the hazards associated with the different activities.

**Figure 4:**
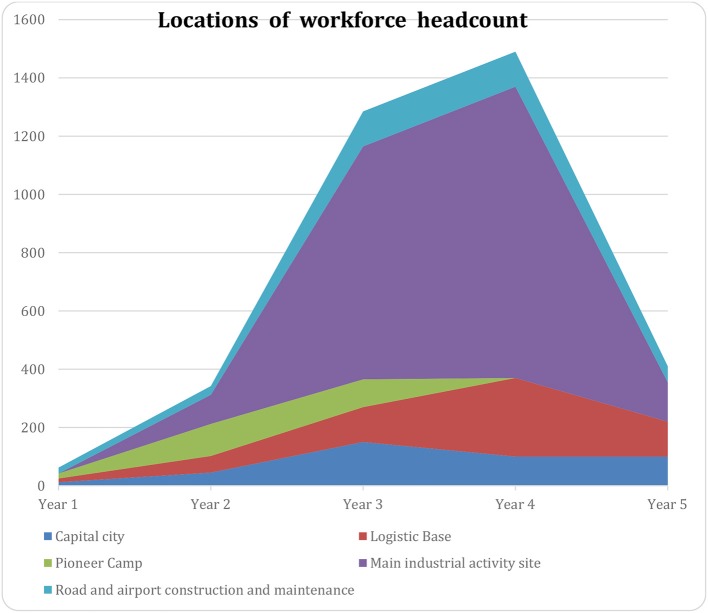
Example of the variation of location of workforce during time (X axis: time, Y number of persons on site).

An example of such a curve is in Figure 4:

**Project timeframe and locations** have major implications for the designing of health support. They are the essential data to be considered when implementing **Site Clinics** and First **Aid Stations**, as well as the positioning of the **Chief Medical Officer** or the **Medical Coordinator**, if any.

### Remoteness of Sites—Evacuation Vectors and Delays

Another preparatory task is to carefully study evacuation time from the site to the relevant healthcare facilities. This must encompass **ground transportation**, **air transportation**, and **cumulated delays** caused by patient transfer from stretcher to bed, stretcher to stretcher, clinic admission, etc…

Sometimes, the situation is unequivocal and the picture is simple. In other cases, various scenarios need to be considered involving many different pathways.

Here are two examples: a simple case (two scenarios) and a complex case (five scenarios) (Tables 1, 2):

**Table 1:** A simple case with two scenarios of an evacuation vectors and delays.

	**Scenario 1 Worksite Track—asphalt road junction City 1 hospital (no CT scan)**	**Scenario 2WorksiteTrack—asphalt road junctionCity 2 hospital (CT scan)**
Track from Site to asphalt road	40 min.	40 min.
Track—asphalt road junction to City 1 hospital	2 h	
Track—asphalt road junction to City 2 hospital		3 h
Total	2 h 40 min.	3 h 40 min.
Track	Ground transportation	
Asphalt road	Ground transportation	

**Table 2:** A complex case with five scenarios of an evacuation vectors and delays.

	**Scenario 1 Worksite City 1 Airport City 3 Clinic**	**Scenario 2 Worksite Site Airstrip City 3 Clinic**	**Scenario 3 Worksite Camp Clinic City 4 Hospital**	**Scenario 4 Worksite Camp Clinic City 4 Airport Repat. Europe**	**Scenario 5 Camp Clinic City 2 Clinic City 4 Airport Repat. Europe**
Worksite—Camp Clinic			10–60 min.	10–60 min.	
Camp Clinic—City 2 Clinic					1 h
Worksite—Site Airstrip		10–60 min.			
Camp Clinic—City 4 Airport				1 h 30 min.	
Worksite—City 1 Airport	2 h 30 min.				
Camp Clinic—City 4 Hospital			1 h 30 min.		
Damage control surgery at City 2 Clinic					Surgery time
City 2 Clinic—City 2 Airport					20 min.
City 1 Airport—City 3 Airport	1 h 30 min.				
Site Airstrip—City 3 Airport		1 h 30 min.			
City 3 Airport—City 3 Clinic	30 min.	30 min.			
City 2 Airport—City 4 Airport					2 h 40 min.
City 4 Airport—Repat. Europe				3–3 h 30 min.	3 h
Total	4 h 30 min.	2 h 10 min−3 h	1 h 40 min−2 h 30 min.	4 h 40 min−6 h	7 h + surgery time
Airstrip or Airport			Air transportation		
Clinic, Hospital, Airstrip or Airport, City			Ground transportation		

(Scenario 5 for the complex case, corresponds to the first flow chart for the trauma patient in section Flow Charts).

### HSE Policies and Documents

A comprehensive study of further documents is necessary to complete the preparatory work. These are:

- **Operator HSE policies** and documents;
- **Subsidiaries HSE policies** and documents;
- Relevant **local legislation**.

The Health Plan must comply with these documents. If amendments are necessary, the earlier the discussion takes place, the better.

### Audit/Study Terms of Reference

Finally, before starting to work, the auditor/consultant must have at hand a document in which the **Terms of Reference** of the audit are clearly described.

This usually takes the form of a bullet-point list including what the client (the company missioning the audit/study) expects as the outcome of the consultancy/study.

Drafting a **Health Plan** is sometimes not even mentioned, when the company does not realize what a powerful tool a Health Plan is. It is part of our job to educate the auditee and demonstrate that such a document, assembling all health-related issues, and evolvable and amendable, is the best way to make sure the audit brings solutions, instead of stockpiling problems.

The wording of these Terms of Reference may vary from case to case, in emphasizing some particular points. **Emergency Preparedness Review** is one of these possible labels. It does not change the methodology exposed here that much, because:

- A complete Health Plan will, of course, address Emergency Preparedness, and;
- Topics sometimes considered as “not that urgent” (such as hygiene conditions of collective sanitation and catering) may turn out to be harmful to a very large number of people very quickly; Hence, ought to be included in the review.

## Risk Assessment

### Approach

In undertaking an “Industrial Risk Assessment,” the engineer seeks to identify all possible causes of industrial accidents. One example of a publication about such accidents, listing processes, related incidents, accidents is: “*OSHA Major Group 13: Oil and Gas Extraction”* (8). In this list, rig collapse, fire, explosion, high pressure release, etc. are among the major accident-related hazards to health.

In relation to clinical features, a great number of mechanisms and circumstances may occur: direct or indirect traumas, falls from height, traumas induced by acceleration/deceleration, heavy load handling, and lifting, etc. Worth being noted about a particular circumstance, an explosion is typically a multiple accident, since the patient will often be a victim of traumatism(s), burn(s), blast effect(s), and multiple wounds.

For the doctor however, it is not necessary to distinguish between the industrial causes of accidents if these scenarios all result in trauma patients presenting the same clinical features, i.e., if the required organizational and medical response means are similar.

The study therefore addresses risk assessment from the perspective of the nature of the injuries as opposed to the nature of the events that caused the injuries. Specifically, the assessment considers:

- Injury Categories;
- Populations at Risk;
- Location issues.

### Injury Categories

Injuries are categorized as follows:

- Traumatic Accidents;
- Industrial exposure;
- Climate;
- Epidemiology.

#### Trauma

Broadly speaking, worksite activities are liable to cause injuries from throughout the entire trauma spectrum, including:

- **Simple traumas** (simple wounds, bruising, sprains, dislocations, slipped discs, simple fractures…) which do not affect the vital prognosis;
- **Multiple traumas** multiple injuries of varying topography which have an impact on the vital prognosis in the absence of resuscitation prior to hospital admission;
  Traumatisms include:
- Traumatisms by shock wave (**blast syndrome**) whether by blast effect (air-borne blast wave), or transmission by solids (solid-borne blast wave) or liquids (liquid-borne blast wave) causing tracheo-pulmonary, cranial, skeletal or abdominal damage;
- Traumas caused by compression (**crush syndrome**);
- **Noise** may cause acute auditory traumas and in the long term, cochlear damage;
- **Vibrations** may cause spinal, vascular, or parenchyma injuries of varying topography;
- **Electric currents** may cause an electrification incident which may cause immediate (ventricular fibrillation, burns,) or delayed (deep injury, coagulopathy, etc.) clinical patterns;
- **High intensity lights** (welding arc, laser…) can cause keratitis (cornea injury) and burns to the retina;
- **The ergonomics** of the workstation, notably in the case of repetitive movements or prolonged incorrect posture, can be associated with muscular or skeletal diseases, notably inflammatory. The length of assignment on site and the pace of work, when excessive, can lead to an increase in the risk of accidents and have repercussions on neuro-psychic and cardiovascular conditions.

#### Industrial Exposure

The study will identify and itemize the various expectable industrial exposures for all industrial activities. Worth mentioning, and recurrent on remote industrial sites, are:

- **Hydrogen Sulfide (H2S)** at Oil and Gas exploration and production sites;
- **Hydrogen Cyanide (HCN)** and/or **Mercury (Hg)** at gold mining sites;
- **Ionizing radiations** during construction phases.

#### Climate

Whether hot or cold, extreme temperatures can harm health. Hygrometry is to be considered as well.

- **Infection of the eyes and respiratory tract** may result from exposure to hot, dry, and dusty air;
- **Heat exhaustion** comes with **dehydration** when hygrometry is high and water intake is not adequate;
- **Heat stroke** is a severe self-deterioration that may occur during heavy work or recreational sport activities;
- **Sun exposure**: prolonged exposure to sunlight carries a significantly increased risk of sun-damaged skin and various skin cancers, the most serious of which is malignant melanoma. North European people with light white skin are a high-risk group;
- **Frostbite** may occur when the Wind Chill factor goes below −30°C (−22°F).

#### Epidemiology

##### Location approach

This is the classical epidemiological study of a region, identifying and itemizing endemic and epidemic diseases, as well as the contamination mode (water borne, vector borne, inter-human, etc.). Not extensively described here**, this chapter is usually the largest** in a risk assessment addressing remote industrial sites devoted to basic materials.

As these sites are very often located in areas where endemic diseases are common, this chapter of the risk assessment will be the reference for immunizations, preventive chemoprophylaxis, and personal protection against endemic diseases. The epidemiologic landscape in some commodities-rich countries is an ever-changing picture, for resistance to antimalarials as well as germs prevalence. Consequently, updating knowledge and recommendations is a duty of doctors and HSE officers, all along the successive amendments of the Health Plan (9, 10).

##### Population approach

Studying the medical aspects of the population at risk is different from the headcount and its breakdown as exposed in section People on Site.

The purpose here is to identify which risk renders more vulnerable which subset of people on site.

- **Cardio-vascular risks among expatriates** over 45 years old are medically well established. From this, the recommendation of a pre-employment medical examination including an ECG and, if relevant, a stress test for expats (male over 45 and female over 50) with a minimum of one extra risk factor may be deduced.
- Frequently, the **local population** suffers from harsh living conditions and medical care is not available to many. This means that common chronic diseases such as high blood pressure, diabetes, vision impairments, precarious dental status, and gastric disorders, to name but a few, are very frequently not identified and/or treated. Given this, and the increasing prevalence of infectious diseases, should a significant part of the workforce be hired from the region, potential employees may have a significantly poorer health profile than the expatriate pool.
  From this may be drawn two conclusions:
  ○ The recommendation of a **medical pre-employment check** for assignment for local workers (see section Pre-employment Check);
  ○ The expectation of a volume of daily consultations **exceeding** the usual average of **2% of the workforce** (see section Expected Routine Activity Volume)

#### Traffic Accidents

As Pre-hospital Intensive Care facilities are usually quasi non-existent in remote areas, a traffic accident may lead to life-threatening situations, because the condition of a severe trauma patient is always deteriorating. The time needed for Project emergency medical personnel to reach the scene may be long.

It is understood that the availability of transmissions (cell-phone or VHF/satellite coverage of logistic convoys and road liaisons) is an important issue, to be addressed in the logistics set-up.

#### Criminal Actions

Kidnapping is an eventuality many fear. It has no specific implication for health support, except that it requires from the doctor on site a tactful approach and skills in psychological support. This should normally be properly performed by any experienced site doctor.

## Existing Health Facilities

### Assessment Method and Grids

For assessing health facilities, the auditor will have to optimize a visit that, usually, does not exceed **half a day**. **An appointment** must have been previously arranged. The **network of personal relations and knowledge of the structures** by the auditing entity is here essential. The contact person (working for the health facility to be audited) in charge of guiding the audit may be a doctor or some administrative/commercial personnel. Whoever it is, the approach must be tactful and constructive:

- Overly intrusive behavior will undoubtedly close doors and deprive the audit of essential information;
- Conduct too “shy” may not allow one to see what it is necessary to see.
  This exercise of “digging” the data out of such an investigation is one of the most difficult tasks needed to successfully construct the final Plan. Throughout the visit, whatever the order in which the various topics are addressed, information must be gathered targeting three main concerns:
  - Practical and technical capabilities of the structure (equipment, skills, hygiene…);
  - Care pathway, particularly in emergency;
  - Availability of contacts (suitably 24/7) for admission and clinical/administrative follow-up.

The possession of detailed grids is obviously an asset for the auditor. Displaying such grids is not within the scope of this presentation.

However, one point should be very clear: **grids are not the silver bullet** with which everything can be obtained. Having very detailed grids (being easily found everywhere) may lead to the feeling that virtually anyone could perform the audit, simply checking the boxes one by one. This is not true. The auditor needs to understand how the facility is functioning and is managed. In-depth experience of how a hospital works is absolutely necessary. Some small details may tell a lot (e.g.,: having a look around an intensive care room).

Finally, some pictures may be taken, with the approval of the contact person, to be added to the report. Obviously, these pictures (no more than 2 or 3 per facility), should focus on points the final commentary intends to highlight.

### On Site Facilities

These may be of very varying sizes and capabilities, from a small Rig clinic, to a large site main clinic. In addition to actual equipment and personnel skills, the audit must check the relevance of the clinic sizing and capabilities, bearing in mind that:

- The **size of the clinic** must be related to the volume of **persons on site**;
- The **emergency capabilities** must be related to the **site remoteness**: the more remote the site, the more sophisticated must be the intensive care capabilities. This is inferred from the tactical reasoning for Medevacs (see section Pathophysiology and Logistics).
  Let us consider two intentionally stereotypical examples:
- A very large oil and gas construction site, say in the Middle East, as close as 50 km from a major city endowed with state-of-the-art hospitals and reachable by an asphalt highway within a 30 min. drive, will need a large routine medicine capability for all the minor work-related injuries and minor illnesses that it is preferable to treat instantaneously onsite. On the other hand, an advanced First Aid team and fast evacuation ambulances will be enough for emergency cases, as it would not be logical to “recreate” a makeshift intensive care facility so close to a more capable one.
- A small exploration site, far inside a huge country, say at minimum 4h00 from the first basic hospital, will need a small routine medicine capability (related to the volume of persons on site), and a somewhat sophisticated intensive care capability and, the case being, a medical escort available, so that acute patients may reach a stabilization facility with appropriate on site and *en-route* treatment.
  Finally, as detailed in section Expected Routine Activity Volume, **a rule of thumb for daily site clinic activity is: 2% of the workforce**. That means that as many as “2% of the workforce” visit the clinic each day. This is considered to be all-included: if a worker comes back twice a day for a dressing change, each visit counts as one. Though this is purely empirical, experience shows it is a very reliable finding, useful for:
- **Pre-conception** of clinic sizing, when volume of personnel is known and health support not yet defined;
- **Afterward audit**: if the daily volume of consultation exceeds the “2% rule,” look for a reason. Usually, an excess of consultations for chronic pathologies that should not be seen onsite is the culprit. Hence, the pre-employment check must be reinforced.

### Local and Regional Backup Hospitals

In auditing local and regional backup hospitals, the main question to be answered is: how will they be integrated in the Health Plan, particularly with respect to stabilization capabilities. In low healthcare level countries, these hospitals will usually have a low level of equipment and hygiene.

However, **damage control surgery** is generally possible, as elementary procedures are usually what these facilities do as everyday practice. The underlying problem is about **anesthesia practices and protocols** which are as basic as the general healthcare in these structures (i.e.,: IV Ketamine Hydrochloride and Opioids, without endotracheal intubation). Hence, it is possible to consider having a **temporary reinforcement** of local hospitals' anesthesia skills by the **Project's doctors or anesthetist nurses** (bringing their specific equipment, including monitoring devices) at the moment of an evacuation to these hospitals. This gives the “best of both worlds”: **modern-like anesthesia and neighborhood experienced basic surgery**. In this case, a second leg evacuation right after surgical stabilization is recommended, so that the patient can be referred to a higher level of healthcare. Another benefit of this set-up is **collegial decision-making** (by local surgeon and expatriate doctor) about whether or not to proceed quickly to a damage control surgery procedure. Experience shows that, sometimes, the local surgeon is dubious about starting such an invasive procedure on an expatriate, being wary about possible consequences in case of a subsequent fatality. This is understandable knowing that the situation is exceptional in the context. Nevertheless, some regional hospitals may perfectly carry out emergency surgery procedures on their own, without any exterior help.

For intensive care (as opposed to Trauma Intensive Care), the picture is somewhat different. Local and regional hospitals usually lack sophisticated hemodynamic management devices and laboratory equipment which are necessary to treat, say, a septic shock or cases of that kind. Stabilization facilities must be found further afield.

As far as Acute Coronary Syndrome is concerned, local hospitals generally do not have appropriate Cath Labs and experience. A long-haul flight is often necessary to reach such locations.

Finally, ancillary investigations for routine medicine (x-rays, basic lab tests,…), as well as specific referrals (ophthalmology…) are usually available and will be the main source of interaction between the site clinic and the neighboring hospital(s).

### Local and National Health Authorities

Some formal but compulsory paperwork is unavoidable, and will be adjusted through a visit to local health authorities:

- The site clinic being a new health facility to be created in the considered administrative area, authorizations, and applications are sometimes required;
- Occupational health national regulations will apply to local workers. Though usually not very strictly enforced in a country's everyday practice, the start-up of a large industrial Project is usually a perfect moment for authorities to demand enforcement of health policies.
- Occupational health for expatriates is usually managed early, prior to assignment. This point is obviously something to clarify with national/regional health authorities in order to avoid later misunderstandings;
- Ambulances are also subject to specifications and regulations. Should the Project hire or acquire ambulances, compliance with these regulations will need to be verified through contact with health authorities.

### Evacuation Facilities—Distant Hospitals

There is nothing special about assessing distant evacuation facilities, also called “nearest centers of medical excellence.” Everything in these facilities must be available for stabilization (if not obtained previously), resumption or finalization of healthcare performed beforehand, follow-up, and final treatment if necessary. Usually at the highest international standard, these structures are most of the time privately funded. Hence, the need of an authorized person from the Project to accompany the technical auditor, to settle financial aspects.

### Drug and Blood Supplies

It is usually impossible to manage a blood bank at a remote site. The audit shall determine if the available public or private blood banks in the country are effectively delivering secure products. The audit must find out how blood is collected (only from volunteer donors?) and screened according to international standards. Typically, family members donate blood to assist a relative. In companies, co-workers usually offer to donate blood as required. In any case, blood transfusion is, as everywhere, to be strictly limited to life-threatening situations. Another option is to ask a repatriation company to bring blood (from its departure country) on-board the inbound flight.

Drug supply is unreliable in low healthcare level countries, and fake drugs are common. A safe drug provision is usually part of the contract addressing site health support.

## On Site Health Support Implementation

### Expected Routine Activity Volume

**A rule of thumb for daily site clinic activity is: 2% of the workforce**. That means that as much as “2% of the workforce” would visit the clinic each day. This is considered all-included: if a worker comes back twice on the same day for a dressing change, each visit counts. Though this is purely empirical, experience shows it is a very reliable finding.

### Routine Care Health Personnel and Emergency Medical Professionals

As stated in section On Site Facilities:

- The **size of the clinic** (for routine care) must be related to the number of **persons on site**;
- on the other hand,
- The **emergency capabilities** must be related to the **remoteness of site**: the more remote the site, the more sophisticated the intensive care capabilities must be. This is inferred from the tactical reasoning for Medevacs.

Consequently, the headcount of routine health care personnel and emergency medical professionals must be adjusted accordingly. These organizations may vary from very basic teams to larger charts including, for example, besides doctors and nurses, anesthetist nurses, ambulance drivers, lab technicians, room boys, administrative agents, etc…

### Camp Clinic(s)—Layout and Equipment of the Premises

Again, based on the findings for tactical reasoning and expected activity volume, camp clinic layout, as well as premise design and equipment come as the conclusion to the preparatory work of the study. Of course, foreseeing this design early, jointly with HSE teams, during (e.g.,) the “conceptual studies” phase of the Project is the best way to proceed, preventing costly recalibrations later on.

### Advanced First-Aiders and First Aid Responders

HSE rules of the head company usually determine how much (what percentage) of the workforce must be trained for Basic First Aid and Advanced First Aid. If not, an example is displayed in the table here below:

**Table:** 

	**Execution personnel**	**Management personnel**
Advanced First Aid	10 %	5 %
Basic First Aid	30 %	15 %

*These percentages are somewhat optimistic and may be amended, considering personnel turn-over which tends to inflate the total head count*.

This is directly under the responsibility of HSE officers and managers. But, being the first link of the rescue chain, a First Aid Responders organization (sometimes appearing as a Site Fire and Rescue organization) benefits greatly from close collaboration with doctors in charge of the Project. In addition, Primary Plans involving First Aid Responders should be written in collaboration by HSE and doctors. Finally, doctors will participate in First Aid Training and Emergency drills.

Where there is a high probability of industrial incident, the proportions given above should be appropriately increased.

### Emergency Rescue Team(s)—Crash Bags

The tasks and capabilities of Emergency Rescue Teams are defined by the Project HSE team. It is important that the content and convenience of crash bags be designed jointly with Project doctors.

### Primary Plans and Contingency Plans

Who takes what actions in response to a given pre-defined situation is the core of a Concept of Operations. This may be also labeled as a **Primary Plan**.

A “**Contingency Plan**” is in most cases used to specify an inventory of existing material and human resources for Health Support.

Insertion of these plans in the general Health Plan is detailed in section Fundamentals for Designing a Health Plan.

### Medical Coordination—Integration in the HSE Command Chart

A large Project may benefit from medical coordination, specific or shared with other Projects. The medical coordinator (usually the Chief Medical Officer) will arrange medevacs with assistance companies and make sure there is coherence between the medical case and the tactical reasoning for medevacs.

It is also a duty of the medical coordinator/Chief Medical officer to amend and update the Health Plan according to ever-changing recommendations for immunizations, preventive chemoprophylaxis, and personal protection against endemic diseases.

### Emergency Preparedness

Emergency preparedness consists in:

- First Aid Training
- Implementation of Remote Damage Control Resuscitation methods (3) and up-to-date Cardio Pulmonary resuscitation techniques (11) all along the rescue chain, from First Aid responders to site doctors and, the case being, medical escort personnel. These techniques include (but are not limited to) the use and practice of:
  ○ Automated External Defibrillator;
  ○ Hemostatic dressings;
  ○ Tourniquets;
  ○ Minimal use of crystalloids and artificial colloids;
  ○ Permissive hypotension (except for Trauma Brain injury patients);
  ○ Tranexamic Acid.
- Drills
  There is a precise methodology for preparing and carrying out an exercise (drill):
  ○ Define the target to be tested (a primary plan of evacuation, the command chain, alert, and vector(s) triggering procedures, a whole site…;
  ○ Identify the scale and impact of the simulated event;
  ○ Write a scenario with a realistic timeframe;
  ○ List constraints;
  ○ Organize debriefings, analyze results and issues, and infer corrective actions (which are not punishments)

### Mass Casualties Plan

There are well established rules for how to manage a mass casualty event. They are based on the classical three step procedure: **collection, triage, and evacuation**.

However, recent developments in war casualty care are trending toward a more “compact” scene management: setting up of a casualty **muster point**, Simple Triage and Rapid Treatment (**START** concept) at this point, and **evacuation** directly from this point.

This last operational concept is directly inferred from military necessity when under fire: there is no time to implement a field medical facility for a comprehensive triage. The priority is to seek shelter, save lives, and move fast.

Nowadays, civilian Emergency Medical Services combine these two concepts, according to the circumstances. For a large housing fire, for example, they will respond more classically with an advanced triage facility located a short distance from the hazardous area. For a mass shooting, the similarities with a war situation lead to responding with the START concept.

In any case, before any advanced healthcare, casualties must be removed from the hazardous area. The questions are: where to, and by whom?

- Where to: **Prior zoning** of the industrial site is essential here. This is usually under the HSE officers' responsibility. Many different types of zoning can be observed: Red, orange, and green zones; exclusion, controlled, and support zones; Pressurized shelters (for gas hazards) and open-air zones;…The Mass Casualty Plan will display these zones and the subsequent initial movement out of danger areas.
- By whom: Obviously, **fellow workers and First Aid Responders** will be in charge of this first step. They have to handle casualties and get them to a safe location, while performing simple life-saving maneuvers: applying wound compression or a tourniquet, having the patient lie or sit, immobilizing a limb, possibly giving Oxygen…

Next, the following steps of the **Mass Casualty plan** are undertaken, with available logistics coming into play. Of course, an attached **Contingency Plan** should display spare healthcare equipment stored for use in this kind of event.

A Mass Casualties Plan may be very specific to a particular hazard (e.g., a toxic gas leak over a construction site) with **an intentionally enabled treatment facility** (e.g., multiple Oxygen or air outlets cascade).

Finally, the evacuation phase is different from what the Tactical Reasoning indicates for a single patient. Personnel categories and status are again crucial here, as the patients will **ultimately be moved consistently with their “company of origin”** (head company, contractor, sub-contractor, etc.) usually after a first **leg move linked to the pathology** (damage control surgery for serious traumas, a medicalized waiting zone for poisonings…).

### Pre-employment Check

As shown in section Approach, a standard evaluation of daily clinic activity is: **2% of the workforce visit the site clinic every day**.

If, when auditing an existing site clinic, the average daily number of consultations stretches far above this figure, it means that something is going wrong. The “usual suspect” is to be found in **local workers suffering from chronic diseases** that should not be found at a remote industrial site, such as: diabetes mellitus, hypertension, dental impairments, etc.

To avoid having to rectify this situation later, a **pre-employment check** must be organized prior to assignment for all newcomers including local workers. In developing countries, the occupational health organization is usually poorly equipped, scarcely funded and sometimes non-existent. Consequently, a network of **diagnostic facilities is to be set up and contracted** for the purpose of checking basic vital parameters and health condition status (blood pressure, ECG, blood sugar, creatinine, dentition, vision, tuberculosis immunization, stool parasites…) Preferentially located close to recruitment centers (i.e., in large cities), this check-up facility (or network of facilities) is usually simple to organize.

On the other hand, **general medical certificates** testifying to a standard medical examination and bearing only the mention “fit to work,” and not displaying any parameters measured at a trustable facility, **are unreliable**.

Comprehensive lists of what such a pre-employment check must cover are numerous and easily available. Displaying them is not in the scope of this publication.

**Pre-assignment check of expatriates** is oddly not always satisfying. **Expatriates (including TCNs) have to undergo the same pre-assignment medical check-up as locals**, under the responsibility of their employer, performed by competent medical providers in modern-like countries

As remote industrial sites are very often located in areas where endemic diseases are common, immunizations as well as preventive chemoprophylaxis and personal protection against endemic diseases must be enforced during these checks (and all along the lifespan of the Project Health Plan).

## Contracting strategy: Subsidiaries, Contractors, and Sub-Contractors Personnel health support

### Necessity of a Contracting Strategy

A large Project usually involves many participating entities such as: the head company (in charge of the Project), subsidiaries, contractors, consultancies, recruitment agencies, etc…Also, throughout the entire set of human resources, different administrative and civil statuses may be encountered:

○ Corporate—employed by the head company or a subsidiary;
○ Contractor—employed by a contracted company;
○ Consultant;
○ Sub-contractors;
○ Local or expatriate;
○ etc…

On large Projects, contractors called EPC (Engineering, Procurement, and Commissioning) usually participate, with a large body of work to deliver, an extended scope of industrial responsibilities and a high headcount. These EPC companies in turn contract other companies (sub-contractors) with multiple sub-contracts.

These contractors and sub-contractors may want to maintain their own camps with collective catering and sanitation facilities involving varying levels of hygiene. Sometimes, they wish to have their own health support.

In case of a large volume of expected EPC personnel (leading to multiple sub-contracts), this can prove difficult to manage, as it yields a multitude of small site clinics with very varying capabilities and technical levels. In case of a serious medical emergency, small sub-contractors do not have the capability to perform pre-hospital intensive care and organize a Medevac.

It is important for the head company in charge of the Project to establish a strategy describing roles and responsibilities, including how the health support load is distributed throughout the Project's participating entities—in other words, providing an answer to the question: “who is paying for the health support provided to each category of personnel in the defined responsibility perimeters?”

### Contracting Strategy

#### Definitions and Principles

The contractual strategy determines the roles of each person, specifying how and by whom routine and emergency healthcare are administered to personnel.

For the contractor's personnel, different options may be chosen by Project management. Among these are:

- **Relying entirely on (sub)contractors** for the health support of their personnel. As described above, this leads to a multitude of small site clinics and a lack of capability to adequately respond to a life-threatening situation.
- **Having a global field health support** to which each contractor shall negotiate access. This is the most reliable option as it gives the head company (in charge of the Project) full control over quality, capabilities and outcome of any healthcare. Financial conditions of this access are to be precisely specified when contracts are signed. This may be difficult to impose on every contractor at the time of contract negotiation. In this case, the head company has the entire responsibility of medical care.
- Some (sub)contractors or individuals may have a **private repatriation insurance or contracted assistance company**. Each (sub)contractor must have its own assistance-repatriation cover as well as consultants. This must be imposed in the contract. It is vital that this be declared at the very beginning of any operation by them, and this data must be part of a **Contingency Plan**. Then, at the moment of an evacuation, immediate contact can be initiated between the medical/logistic center of the repatriation company and the health personnel on site/Project senior medical coordinator.
- To deal with asymmetries among the Project participating entities, an option is to contract a Project Membership product, sold to cover a population on a specific client site in which eligibility for provision of services is related to the specific site, regardless of the origin of the patient.
  The best contractual strategy has to be a mix and match from these options, making the health support consistent with Project management goals.
  Creating a comprehensive and reliable contracting strategy for healthcare provision, covering every employee, is probably **the most difficult task** in designing Field Health Support.
  Failure to do this proves to be the main source of delays and mismatches whenever a medical emergency occurs, with very serious and costly consequences.
  The recommendation, to avoid these issues, is to have **medical engineering** and **industrial engineering** proceeding in concert. To achieve this:
- **Industrial contracts** should have a **health exhibit** in which the health responsibilities of (sub)contractors are specified. These may include, but are not limited to: primary care on site, clinic or health personnel provision, evacuation to a pre-determined hospital, and hygiene standards to be enforced.
- The **Project Health Plan** is the document, clearly communicated to all parties involved, which presents the strategy, and will remain the permanent reference document referred to in contractors site HSE Plans or bridging documents.

#### Roles and Responsibilities for Field Health Support

- **Primary care assistance provider:** This provider is present at an isolated site with workers and is in charge of supplying primary care/emergency care on site, and, if necessary, the evacuation first leg;
- **Secondary care assistance provider:** This provider becomes active at a scheduled time, at a point defined as **a handover location**. A handover location is where responsibility of one Tier is transferred to another Tier. A secondary care assistance provider usually operates from a logistic platform of general use for evacuations;
- **Stabilization and handover:** As indicated in section What Stabilization Actually Means, stabilization is a medical term referring to the main clinical feature that is common to all critical clinical situations: a self-deterioration through auto feedback loops;
  The **stabilization point** is the medical facility that has the means needed to stop the self-deterioration of the pathophysiology;
- On the other hand, the **handover point** is the pre-defined geographical location, accepted by all parties involved, where the activity of the primary care assistance provider ends, and where the secondary care assistance provider sends to, or organizes, health support in relay. This point should be predefined in the Medical Emergency Response Plans issued by all parties involved and updated as often as necessary.
- Usually, when sites are isolated, stabilization, and handover points do not coincide. Coincidence would exist only if a big hospital were close to Project site.
- The patient reaching the stabilization point is the target for the Chief Medical Officer (CMO), coordinating appropriate field (intensive) care, and transportation. The patient's route may pass through different handover locations, relying on a chain of different assistance providers.
- **Repatriation:** If the patient is an expatriate, the Medevac:
  ○ May be followed by a repatriation (in a second leg, as soon as possible after stabilization);
  ○ May be repatriation in itself if the relevant stabilization hospital is in a developed country with appropriate-level standards of medicine.

In any case, a file must be opened and kept up to date so that, for any expatriate working on the Project, the name and details of his/her assistance/repatriation provider are known, whether it is coverage by employer/contractor, a personal contract, or any combination of these.

The CMO is the medical contact for repatriation companies involved in the Project. He ensures the quality of the first medical contact and report with the medical dispatcher of a repatriation company. This action is a major step in the carrying out of a quick repatriation with appropriate means and conditions;

These considerations imply that the contracting strategy shall:

- **Identify all parties involved** (primary and secondary care assistance providers, head company, contractors, the contractor's own health service provision, repatriation companies etc…);
- **Define perimeters** for all of them, referring to handover locations that have been previously validated for medical and logistical considerations.

#### Financial Risk and Medical Engineering Perspectives

The financial conditions for contracting a secondary care assistance provider may be labeled as either “access” or “service.”

- An “access” contract provides a warranty of intervention after a minimal fee has been paid. The possible interventions will be charged case by case. This type of contract is usually assorted with a list of “authorized persons” i.e.,: the managers having the authorization to trigger a Medevac;
- A “service” contract is an “all inclusive” insurance program, in which all actions needed are covered by global pre-negotiated fees.
- In practice, the difference between those two forms of pricing may be minimized, as a renegotiation usually occurs when claims reach an unexpected level, or if no-claim bonuses apply.
- In developing the contracting strategy, issues related to liabilities, insurances etc., also need to be addressed. They are clearly beyond the domain of medical engineering, usually being part of the Project financial risk manager's responsibility.
  Financial risk management deals with capital expenditure and pricing options whereas medical engineering, working jointly with industrial engineering, is focused on how routine and emergency care may be delivered while staying as close as possible to state-of-the-art standards in an area where risks are high and medical capacities are low.
  Financial risk and insurance management is also involved in secondary care assistance provider qualification.

### Three Steps of the Contracting Strategy

#### Defining Perimeters

**The first perimeter** is the industrial site itself with its work area, camps, and living quarters. There, primary care is provided by a chain of First Aid responders and a First Aid Station or a site clinic, when available. This perimeter is usually under the responsibility of companies operating on site and their HSE management.

**A second perimeter** is the main site clinic, if not included in the previous care provision, and the medevac first leg.

From this step, the provision of healthcare services may be outsourced to a medical provider.

This clinic usually has an ambulance available, ready to evacuate a patient to a hospital, airstrip, airport, etc. and sometimes may also access to a Company helicopter. This first leg of evacuation may or may not, be at the expense of the industrial entities.

Consequently**, a first handover point** may appear in the Care Organization System at this very first location, such as the “door” of the site clinic, depending on who is in charge of providing the vector and/or the medical escort of this medevac first leg.

Moreover, the tactical reasoning (see section Tactical Reasoning for Medevacs), may result in different evacuation routes, according to the clinical features involved. Routings may also vary with personnel status, since a local employee may be preferentially referred to the national health structures, whereas an expatriate may be directly repatriated or evacuated to the nearest center with modern-like medical capabilities.

This last point is not without an ethical conundrum, as one may desire equal treatment for all. Alas, on-the-ground realities usually yield a harsher distribution of unequal healthcare provisions.

Finally, the medevac first leg may or may not reach a stabilization point (see section Flow Charts). If not, the second leg evacuation will aim to reach the stabilization facility.

Consequently, logistics and pathophysiology sequences may not always coincide, particularly for remote industrial locations.

**A third perimeter** typically concerns where a secondary care assistance/healthcare provider operates from, up to the final destination. This is the domain of assistance companies, long haul evacuation flights, and/or high-level hospitalization capabilities.

**The CMO** (see section medical coordination) must have a global view of these routes, handover points, and facilities' capabilities, and ensure a correct execution of the plans in joint decision making with the HSE officers involved and the medical coordination board of the assistance companies involved.

#### Contracting—Writing Health Exhibits of Industrial Contracts

Three different types of contracts are linked to health support:

- I**ndustrial contracts** in which, along with the main industrial activities, contractors' duties are specified. Health related specifications are gathered in the so-called **Health Exhibit(s)**. These duties may include, but are not limited to, primary care on site, clinic or health personnel provision, evacuation to a pre-determined hospital, hospitalization expenses, etc…;
- Contract(s) between the head company and **the medical provider(s)** in which **primary care and secondary care assistance** are defined and contracted;
- Contract(s) between **industrial contractors** and **the medical provider(s)** in which industrial contractors' duties (such as **primary care** for certain categories of personnel), as specified in industrial contract **health exhibits**, are represented and contracted (exceptions are cases where a contractor provides some elements on its own). Here, the head company may have a right to supervise the choice of the medical provider(s) through a qualification process.

These three sources of health specifications need to be harmonized to make the health support consistent with Project management goals.

#### Monitoring and Enforcement

The **Project Health Plan** is the document communicated to all parties involved, displaying the strategy and being the permanent reference document referred to in contractors' site HSE Plans or bridging documents.

**Assistance/Medical providers qualification:** Assistance/Medical providers may be hired directly or by an industrial contractor. In the second case, the head company should retain the right to valid the offer, according to:

- Health specifications in the relevant exhibits of the industrial contracts;
- Project Health Plan.

It is important that the Project financial risk and insurance management be involved in the assistance setting.

**Projection capabilities**: The Project Assistance/Medical provider qualification process must take into account the local and regional projection capabilities of a secondary care assistance provider. In other words, the evaluation relies for an important part on assessment of whether the handover point(s) is/are suitable and consistent with Project management goals. **The closer to Project sites the handover points are, the easier it is to organize a Medevac to a stabilization point**.

**Dedicated features:** The Project provider qualification process shall evaluate if the offered provision is suitable for the site Emergency Response Procedures, regarding:

- Dedicated, Project-specific operations, and emergency procedures;
- Dedicated alarm/call facilities for the site;
- Dedicated alarm/call medical staff, aware of site specific protocols, and emergency response.

## Hygiene

### Collective Catering

#### Reference Documents

Many reference documents may be found, particularly among representative organizations for Oil § Gas and mining company publications, as well as from specialized agencies.

To cite but a few:

- **Oil & Gas UK** (OGUK former UKOOA) has a record for publishing comprehensive recommendations setting out collective catering, water production, and waste management rules. Although dedicated to offshore installations, the published guidelines encompass environmental health risks and practice: respect, high standards of hygiene, applicable to any remote industrial site. A latest update can be found here: (12)
- **International Association of Oil & Gas Producers** (IOGP former OGP) publishes numerous guides notably to Food and Water Safety: (13)
- The United States Department of Labor **Occupational Safety and Health Administration** (OSHA) enacts standards for labor camps, food handling, and water supply: (14)
- The **World Health Organization** (WHO) regularly updates guidelines for drinking-water quality (15)

#### HACCP

All these guides reiterate the core principles applicable to all types of installations, notably the: “**Hazard Analysis and Critical Control Points**” (HACCP) approach. This is a systematic preventive approach to food safety concerning biological, chemical, and physical hazards in production processes which can cause the finished product to be unsafe, designing measurements to reduce these risks to a safe level.

The HACCP principles are included in the International Standard ISO 22000 FSMS 2011.

On a remote industrial site, a manager usually called the “**Camp Boss**” is responsible for implementing and continuously monitoring the HACCP procedure.

From production to consumption and waste management, a complete food chain is built. The essential points are:

- Production/Purchasing
- Storage/Packaging/Transport onsite
- Reception
- Dry/refrigerated/frozen storage
- Hot/cold preparation
- Cooking
- Keeping foods hot
- Refrigeration
- Reheating
- Serving
- Washing up
- Waste management

Each one of these points is a source of a potential incident which could affect the quality of foodstuffs and lead to a potential collective health problem.

The workflow for this chain is shown below.

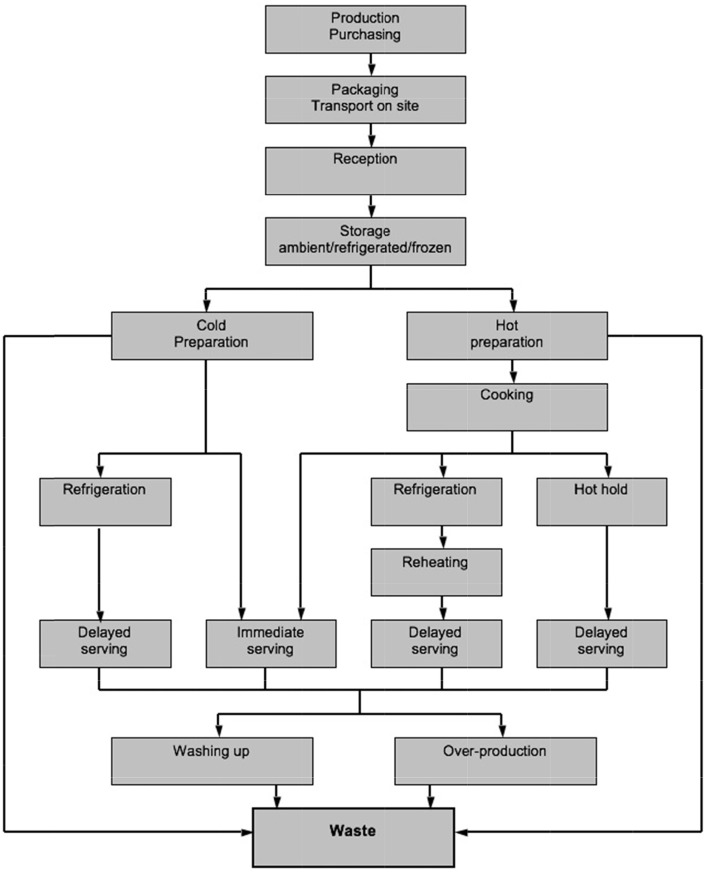



#### Collective Catering Audit

In auditing collective catering facilities, similarly to auditing health facilities, the approach must be **tactful and constructive**. The audit will be conducted jointly with the Camp Boss.

New auditors usually have a checklist in hand while proceeding. With experience, the checklist becomes memorized. It is a current practice, though, to sign the completed checklist jointly with the Camp Boss at the end of the visit.

**A separate hygiene audit report** will preferentially be given “hand to hand” to the Camp Boss before being included in the global site audit report. This way of proceeding enhances trust and proactiveness. In reality, the Camp Boss, immersed in the ebbs and flows of camp management, often perceives an external auditor as unaware of his/her daily struggles, and prompt to focus on minor things. It must be clearly stated, before proceeding with the audit, that nothing is ever perfect and everything is subject to improvement. However, being tolerant about minor misfits linked to a specific site condition does not exclude, and even reinforces, firmness with respect to an unacceptable default.

It is worth noting **that site doctor(s) must regularly carry out such an inspection**, at a minimum on a weekly basis. Apart from an external audit, it is a very important duty of a site doctor, jointly with HSE managers, to monitor camp hygiene, conduct water sample analyses, check sanitation, etc. Hence, a preliminary discussion with the site doctor in charge is to be considered before proceeding with the inspection.

That said, having the checklists and regulations duly available or memorized, here are some complementary tips:

- The whole food chain is to be inspected, with particular attention to **food storage**. Wearing a jacket and spending minutes in refrigerated rooms is always worth the effort.
- When inspecting **kitchens**, it is preferable to conduct the visit before noon, when cooks are at work preparing lunch meals. Failures to observe safety practices are easy to detect at this time, whereas an afternoon visit will often only show a cleaned and tidy after-work kitchen. Particular attention must be paid to hand washing basins, disposable paper towels, chopping boards, waste circuit, and anti-vector (rodents, cockroaches…) measures. A pest control program should have been implemented.
- Inspecting **canteens** must focus not only on cleanliness and availability of hand washing basins, but also on crowding and quality of the lunch time break.
- **Fitness to work of cooking personnel** is a specific topic, to be discussed beforehand with site doctors. Some complementary medical examination is mandatory here, unlike for other personnel categories, such as:
  ○ Hepatitis (A) serology;
  ○ Stool analyses;
  ○ Clinical examinations to detect transmissible diseases, particularly skin, lung, urinary, and digestive diseases;
  ○ Tuberculosis detection;
  ○ …
- **Control meal samples** must be stored (frozen or vacuumed) and kept available for 72 h after every meal has been served. Most catering companies keep the samples about 5 days.
- **Kitchen staff must have dedicated changing rooms with lockers and toilets**;
- Not only must the general hygiene of catering personnel be enforced concerning hand washing, clothing, safety equipment, etc. but in addition **a continuous specific training program** must be established for this very critical category of personnel, addressing:
  ○ Personal hygiene;
  ○ Cleaning and disinfection of the premises;
  ○ Foodstuff storage;
  ○ Food preparation and service;
  ○ The waste cycle;
  ○ Anti-vector measures;
  ○ …

### Collective Toilets and Showers

Sanitary installations, showers, and wash hand basins must be **cleaned and disinfected every day** using chlorine-based products or an equivalent. These instalations must respect privacy of the users (locking doors, curtains in proper status, etc.).

Regular cleaning of the sanitary installations must include the **showerheads**. Given that they produce a spray of hot water, they are a fertile proliferation nest for *Legionella pneumophila* bacteria.

In general, a specific prevention plan for *Legionella pneumophila* must be established, jointly with HSE officers, based on water production specifics of the camp.

### Water

The quality of water for domestic use at a remote site is a critical and large issue. Comprehensive recommendations are available, published by numerous agencies (see above). As for catering inspection, **regular water testing** and quality of water monitoring **is a weekly task**—sometimes needed twice a week—and a permanent concern for site doctors and HSE officers. Here also, a HACCP procedure is to be conducted, addressing:

- Volumes produced (related to the supported population);
- Storage safety;
- Water sources (wells, pumping stations, existing networks…);
- Treatment (desalting, chlorination, filtration, UV sterilization, reverse osmosis…)
- Quality control (sampling points, regularity, camp proofers, complementary lab analyses…)
- Treatment of used water before discharge

### Laundry

All bed linen and clothing, including underwear, must be cared for at the appropriate temperature in the laundry. **Individual clothes washing is to be prohibited**.

The laundry room must be cleaned at all times. A useful tip: **tumble dryer filters** must be checked. Frequently forgotten, these filters collect a very thin cloth dust which is, due to the dryers' internal temperature, **highly inflammable**. Cleaning these filters is not only a matter of concern for hygiene, but also for **fire prevention**.

### Waste Management

Waste management is under the HSE officers' responsibility, jointly with site doctors. Medical and biological waste is usually directly processed (stored and sent to an internal or external incineration facility) by site clinic personnel.

Keywords are: triage, collecting, storage, recycling, or destruction. Adequate containers with tagged labels are necessary.

Here is an example of a waste management plan, summarized in Table 3:

**Table 3:** Example of a waste management plan.

**Waste class**	**Type**	**Tag code**	**Examples**	**Storage**	**Disposal methods**
**Oily Waste**	Liquid	xxx	Diesel, hydraulic oil, lube oil	Drums and Tanks with **Black** Label	Recycling Send to.…
Industrial Waste	Solid	xxx	Paint tins	Bins or Dumpsters with Red Label	Incineration
Industrial Waste	Solid	xxx	Empty metallic drums	Baskets with Red Label	Recycling Send to…
Industrial Waste	Solid	xxx	Acids, solvents, glycol, electrolytes	Tanks with Red Label	Transfer to … for chemical process
Medical Waste	Solid	xxx	Dressings, syringes, sharp containers	Bags and containers with Yellow Label	Transfer to … for incineration
Metallic waste	Scrap metal	xxx	Metal sheets, cables, auto parts, cans	Baskets with White label	Transfer to … for recycling
Non-metallic industrial and office waste	General	xxx	Paper, cardboard, wood, glass, plastics, other inert material	Bins with Blue Label	Disposal to … waste landfill
Food	Food	xxx	Food **Packaging excluded**	Bins with Green Label	Incineration in camp burning pit

Sanitation systems have their own waste processes for human feces and sewage wastewater. Except for temporary camps, these are usually Container Based Sanitation (CBS) or Onsite Sanitation with septic tanks and onsite sewage facilities.

In temporary camps, pit latrines, bucket toilets, container-based toilets, or chemical toilets are the usual solutions encountered.

### Pest Control

Pest control, particularly for rodents, is another topic of joint management by HSE and clinic teams. An important point to check during the audit is: **How are the disposal and litter bins areas maintained**. These areas attract many unwelcome pests if not maintained properly.

Maintaining these areas properly depends on **all of the personnel being duly instructed and motivated**. Pest control is an excellent topic for the routine briefings that are usually held regularly in camp daily life. If people do not realize how important it is, it may be stressed that “**waste attract rodents, then rodents attract snakes!**” Using this aphorism usually increases everybody's motivation, even though improper litter management risks go far beyond the possible presence of snakes.

Efficient **rat proofing** relies on precise knowledge and needs specific tools and tricks. Guides on the topic are easily available.

**Mosquito bite prevention** is another important point, particularly in vector-borne diseases areas (malaria…). There are well-known specific domiciliary and peri-domiciliary measures to be taken. The relevant aspects of an industrial site are all mechanical structures that may gather rainwater and, hence, be **larvae nests**. Sometimes, at large construction sites, it is impossible to have all of them treated with insecticides. Fighting vector-borne diseases then relies on a multi-target action: mosquito fighting, chemoprophylaxis, early detection, and early treatment when available.

## After the Audit/Study

Having visited the site, held interviews with managers and health personnel, gathered all background information (medical facilities, transportation providers…), and eventually worked over the tactical reasoning with HSE officers, it is time to **write a report**.

Such a report will be a useful piece of work for the Project if:

- It is **easily readable**, with partial and general conclusions providing actual help for managers to **make decisions**;
- Its organization and presentation allow it to be **directly transformable into a Health Plan**.

### Writing an Audit/Study Report

The organization for the report proposed below has the advantages of:

- Offering clear and practical **proposals** to diverse types of readers, according to their hierarchical level, i.e.,: **executives officers, line managers, and specialists**. To achieve this, **different degrees of detail** will be presented in the respective parts;
- Developing chapters and paragraphs in a logical way, displaying the **global strategical picture and its included tactical reasoning**. This way, the report is in itself *mutatis mutandis* a draft for a Health Plan.

#### Report Pattern

- Write the main body of the report;
- Have every paragraph concluded with a double bullet point subsection called **Findings** and **Recommendations;**
  Example:
  **Findings:**
  ○ A significant journey by ground ambulance between the Project site and City X *via* a gravel track is clearly not the ideal condition for the transport of the seriously sick or injured
  ○ At the site airstrip, night landing remains to be requested; Air Procedures (meet and greet) must be prepared.
  ○ There is a need for a local VHF loop or alternative such as satellite phones, where cell phones do not work.
  **Recommendations:**
  ○ Consider implementing/providing, earlier than scheduled, an asphalt road between Project site and City X.
  ○ Ask for night landing clearances, and prepare for meet and greet Air procedures at the site airstrip
  ○ Provide isolated Project worksites with satellite phones or a VHF loop.
- Copy and paste **all the Findings and Recommendations subsections into a table** located **after** the main body of the report. Add **priority levels** to the recommendations;
- Write a one **page summary** of these finding and recommendations, highlighting **context and forward guidance**, label it **Executive Summary**, and place it **at the front of the report**, before the main body text.

The report has now the following pattern:

1. Executive Summary;
2. Main body of the report, unfolding the reasoning, with Findings, and Recommendations at the end of every paragraph;
3. Table of Findings and Recommendations with priority levels;
4. Appendices

It is conceivable that executive officers will read just the one page Executive Summary, say “yes” or “no” or “how much does it cost?” to relevant points and pass on the report to their HSE managers. Then, the high level HSE managers will surely examine in detail the table summarizing the findings and recommendations, and go into the main body of the report only for one or two specific topics to be investigated. Finally, the medical specialists and field HSE managers, in charge of writing the Health Plan, will read everything and use the main body of the report to directly draft the Health Plan.

#### Report Organization

Developing the global reasoning suggests having the following organization of contents:

Executive Summary

Abbreviations, acronyms, and terminology

1. Introduction
  1.1. Objectives, output, and complications
  1.2. Terms of reference
  1.3. Basis of assessment
    1.3.1. Health facilities reviewed
    1.3.2. Persons interviewed
    1.3.3. Reference documents
2. Risk assessment
  2.1. Approach
  2.2. Injury categories
    2.2.1. Trauma
    2.2.2. Industrial exposure
    2.2.3. Climate
    2.2.4. Epidemiology
  2.3 Population at risk
    2.3.1 Medical aspects
    2.3.2 Person on site—Volume and categories
  2.4 Remoteness of sites—Evacuation vectors and delays
    2.4.1 Ground transportation
    2.4.2 Air transportation
3. Reference backup hospitals
  3.1. Hospital X
  3.2. Clinic Y
  3.3. Distant hospitals for long-haul medevac
4. Complementary background information
  4.1. Drugs and blood supplies
  4.2. Local and regional health authorities
  4.3. Aircraft providers
    4.3.1 Regional charter airlines
    4.3.2 Long-haul air ambulances
    4.3.3 Commercial carriers
  4.4. Airstrips/Airports
  4.5. Medical escort
5. Current site medical support
  5.1. Follow-up of previous audits
  5.2. Camp clinic
    5.2.1. Layout, condition, and equipment at the camp clinic
    5.2.2. Activity and significant emergency cases
    5.2.3. Health personnel
    5.2.4. Ambulance(s)
  5.3. Emergency rescue team and First Aid training
  5.4. First Aid posts
  5.5. Case and document reviews
6. Health support to be implemented (immediate)
  6.1. Health Plan—Concept and writing/drafting
  6.2. Care Organization System—Routine and emergency cases
  6.3. Tactical reasoning for medevacs
  6.4. Medevac flow-charts
  6.5. Primary plans
  6.6. Contingency plans
  6.7. Mass casualty plan
  6.8. Compulsory sanitary reports
7. Health support to be implemented (future)
  7.1. Site clinic (same breakdown as § 5.2)
  7.2. Other health facilities
  7.3. Emergency rescue teams and first aid training
  7.4. First Aid posts
8. Contractual strategy
  8.1. Concept and principles
  8.2. Roles and responsibilities for field health support
    8.2.1. Primary care assistance provider
    8.2.2. Secondary care assistance provider
    8.2.3. Providers’ projection capabilities
    8.2.4. Stabilization and handover
    8.2.5. Repatriation
  8.3 Contracts
    8.3.1 Healthcare/Assistance provision contracts
    8.3.2 Health exhibits to industrial contracts
  8.4 Providers qualification
9. Hygiene
  9.1. Collective catering
    9.1.1. HACCP and inspections
    9.1.2. Food supply
    9.1.3. Food storage
    9.1.4. Defrosting
    9.1.5. Kitchen(s)
    9.1.6. Canteen(s)
  9.2. Collective toilets and showers
  9.3. Water
    9.3.1. Process overview
    9.3.2. Water testing
    9.3.3. *Legionella pneumophila* prevention
  9.4. Laundry
  9.5. Waste management
  9.6. Pest control, mosquito fighting
  9.7. Diet and recreation
10. Summary of findings and recommendations
Appendices

Writing and organizing the audit/study report this way saves a lot of time because **a “copy and paste” version of chapters 2 to 9 is a Health Plan**

### Monitoring, Enforcing, and Amending a Health Plan

#### Compulsory Sanitary Reports

Three types of reports should be required from site health facilities:

- **Reports to local and national health authorities**; Respecting their particular specifications
- **Reports to company(ies) medical department(s)**; If the head company and/or its main contractors have established medical departments supervising foreign operations.
- **HSE reports**; HSE officers may require doctors on site to provide some data and information to their compulsory reporting activity.

These reports, as well as regular site visits, are necessary to enforce Health Plan arrangements and rules.

#### The Health Plan Is a “Living” Document

All along Project life, the Health Plan will evolve.

Head company doctors, doctors on site, and HSE officers will need to **amend** it every time a **new tactical situation** arises in the Project.

Through **audits**, such as one following the methodology described in this publication, the Health Plan will be **shaped and re-shaped** to reflect management goals.

### Health Plan and Project Management

Large industrial companies have a **Project Management process**, which is usually the object of very close attention and a precisely detailed method.

Roughly speaking, a phased approach plans:

- Conceptual studies;
- Pre-project studies;
- Basic and detailed engineering studies;
- Construction and procurement planning;
- Site main facilities construction;
- Commissioning;
- Production;
- De-commissioning and phasing out.

Project technical and financial reviews, statements of requirements, and conclusive documents are developed to conclude each phase and trigger the following one.

It is very important that **a first draft of the Health Plan be prepared as early as the first phase**. Thereafter, continuous work will ensure suitable health support preparation, preventing costly and time-consuming corrective actions that are always necessary when a global strategy and adequate tactics have not been implemented:

- When engineers are busy gathering data to feed their technical concepts, it is also time for the medical engineer to collect useful information for health support;
- When industrial contractors are selected, it is also time to introduce medical and assistance providers into the selection processes;
- When procurement logistics are prepared and triggered, it is also time to take care of equipment and consumables with long delivery lead times;
- Etc.

## Conclusion

Health support engineering for remote industrial sites, in low sanitary level countries where most industrial commodities Projects take place, is a joint work effort by HSE and medical specialists

This review provides a synthesis of the main steps that need to be undertaken to eventually develop a Project Health Plan.

A core part of the conceptual work consists in partnering two fields that usually go their own way: pathophysiology and logistics. Each one may be considered in terms of delays: In life threatening situations, pathophysiology allows for a (maximum) delay before effective stabilization, while logistics dictates a (minimum) delay to reach a stabilization facility. Having these two delays correlate adequately with each other is the expected result. This requires careful study of all the various components, and a methodology described here.

Finally, Health Support implementation according to population on site, contracting strategy, Emergency Response Plan drafting and hygiene review will complete the task and be included in an audit/study report.

An audit/study report must result in conclusive recommendations. Hence, guidance is proposed so that the report becomes a matrix of the Health Plan itself, and will be closed by a summary of findings and recommendations ready-for-use by Project management. In this way, the Health Plan will be launched to gradually evolve and be amended/updated as a “living document” throughout the lifetime of the Project.

These synopses are probably doing injustice to all the nuances of remote industrial site features, but the aim is to get the big picture right.

## Author Contributions

TL was at the origin of the text. AD supervised the drafting process with significant improvement of all authors (TL, SG, AG, and AD).

### Conflict of Interest Statement

TL is employed by AP-HP (Assistance Publique Hopitaux de Paris) as an emergency physician. SG is employed by FRANCE MÉDIAS MONDE as corporate doctors. AG is employed by AXA PARTNERS as Deputy International Medical Director. AD is employed by AP-HP, UVSQ (Versailles University) as occupational health professor, Univ Angers/CHU Angers as an associate member. AD is also employed by Elsevier Masson as editor in chief of Archives des maladies professionnelles et de l'environnement. No other competing interests are needed to be declared.

## References

[1] Wakerman J. Defining remote health. Aust J Rural Health. (2004) 12:210–4. 10.1111/j.1440-1854.2004.00607.x 15588265

[2] Rosser JC Vignesh V Terwilliger BA Parker BC. Surgical and medical applications of drones: a comprehensive review. JSLS. (2018) 22:e2018.00018. 10.4293/JSLS.2018.00018 30356360 PMC6174005

[3] Chang R Eastridge BJ Holcomb JB. Remote damage control resuscitation in austere environments. Wilderness Environ Med. (2017) 28:S124–34. 10.1016/j.wem.2017.02.002 28601205 PMC5608023

[4] Wyer PC Silva SA Where is the wisdom? I - a conceptual history of evidence-based medicine: a conceptual history of EBM. J Eval Clin Pract. (2009) 15:891–8. 10.1111/j.1365-2753.2009.01323.x 20367679

[5] Djulbegovic B Guyatt GH. Progress in evidence-based medicine: a quarter century on. Lancet. (2017) 390:415–23. 10.1016/S0140-6736(16)31592-6 28215660

[6] Huguenard P Capoulade M L'hélicoptère sanitaire. Urgences Méd. (1995) 14:202–6. 10.1016/0923-2524(96)80640-0

[7] Huguenard P Larcan A Noto R. Advance medical planning and disaster situations. Rev Prat. (1988) 38:648–56. 3387869

[8] Major Group 13: Oil And Gas Extraction Occupational Safety and Health Administration. Available online at: https://www.osha.gov/pls/imis/sic_manual.display?id=8&tab=group (accessed December 16, 2017).

[9] Tizifa TA Kabaghe AN McCann RS van den Berg H Van Vugt M Phiri KS. Prevention efforts for malaria. Curr Trop Med Rep. (2018) 5:41–50. 10.1007/s40475-018-0133-y 29629252 PMC5879044

[10] Toovey S Moerman F Van Gompel A. Special infectious disease risks of expatriates and long-term travelers in tropical countries. part II: infections other than malaria. J Travel Med. (2007) 14:50–60. 10.1111/j.1708-8305.2006.00092.x 17241254

[11] Schunder-Tatzber S Dagrenat C Boucif S Issard D Cassan P Baer M. Cardiac arrest at the workplace: results from an international survey about First Aid on Red Cross and Red Crescent Societies and International Companies Network. Resuscitation. (2016) 108:e1–3. 10.1016/j.resuscitation.2016.08.031 27616582

[12] Oil & Gas UK Publishes Offshore Environmental Health Guidelines. Oil and Gas UK. Available online at: https://oilandgasuk.co.uk/product/guidelines-for-environmental-health-for-offshore-installations/ (accessed December 16, 2017).

[13] A Guide to Food & Water Safety – IOGP Bookstore. Available online at: http://www.iogp.org/bookstore/product/a-guide-to-food-water-safety/ (accessed December 16, 2017).

[14] and Health Topics Foodborne Disease - OSHA Standards | Occupational Safety and Health Administration. Available online at: https://www.osha.gov/SLTC/foodbornedisease/standards.html (accessed December 16, 2017).

[15] WHO | Supporting publications to the Guidelines for drinking-water quality WHO. Available online at: http://www.who.int/water_sanitation_health/water-quality/guidelines/drinking-water-guidelines-publications/en/ (accessed December 16, 2017).

